# From Roadways to Wheeze: Child Asthma Associated with Traffic Exposures at Home and at School

**DOI:** 10.1289/ehp.118-a305b

**Published:** 2010-07

**Authors:** Julia R. Barrett

**Affiliations:** **Julia R. Barrett**, MS, ELS, a Madison, WI–based science writer and editor, has written for *EHP* since 1996. She is a member of the National Association of Science Writers and the Board of Editors in the Life Sciences

Approximately 6.2 million children in the United States are affected by asthma, a chronic respiratory disease that is becoming increasingly common in developed countries. Air pollution has been identified as one potential cause for the increase, with proximity of children’s homes to heavy vehicular traffic being a particular investigative focus. However, research results have not been definitive. A unique prospective study has explored the role of traffic-related pollution in causing asthma by estimating children’s exposure both at home and at school against the backdrop of regional ambient air pollution **[*EHP*** **118(7):1021–1026; McConnell et al.]**. The results associate both school and home exposures with new-onset asthma in young children.

The prospective study included 2,497 children who were asthma-free upon enrollment in the Children’s Health Study during the 2002–2003 school year, when they were in kindergarten or first grade. The cohort represented 13 Southern California communities and 45 schools, and the children were followed for 3 years. Parents completed baseline and yearly surveys providing information on demographic characteristics, respiratory illnesses, and risk factors for asthma. Children with physician-diagnosed asthma, symptoms suggesting undiagnosed asthma, or incomplete health symptom data were excluded from the study.

Temperature, relative humidity, and ambient air levels of ozone, nitrogen dioxide, and particulate matter were measured continuously at a central monitoring station in each community. Local traffic-related pollutants were estimated for each child’s home and school using a model that incorporated roadway proximity, local traffic density, vehicle emission rates, and meteorologic variables.

During the study, 120 children developed asthma. Development of asthma was independently associated with traffic-related pollution at school and at home; the authors observed a statistically significant association with nitrogen dioxide and nonsignificant associations with fine and ultrafine particulate matter. However, an overall measure of traffic-related pollution (from both freeway and nonfreeway sources) and estimated exposures to individual pollutants were all associated with a significantly increased risk of asthma regardless of whether the exposures occurred at school or at home.

Estimated effects of exposures at school and home were comparable, despite the fact that children spent less time at school than at home. The authors offer possible explanations for this finding: panting during playtime and physical education can increase the dose of pollutants delivered to the lungs, plus children typically arrive at school during morning rush hour, when pollutant levels may be particularly high.

This study strengthens the evidence that nearby vehicular traffic contributes to asthma development and highlights a critical public health concern given that large populations of children are exposed to traffic-related pollutants at school. Controlling vehicular emissions and planning transportation and urban development to limit exposure to traffic-related pollutants could significantly benefit children’s health.

